# Arginine‐Loaded Nano‐Calcium‐Phosphate‐Stabilized Lipiodol Pickering Emulsions Potentiates Transarterial Embolization‐Immunotherapy

**DOI:** 10.1002/advs.202410484

**Published:** 2024-12-16

**Authors:** Duo Wang, Lei Zhang, Wei‐Hao Yang, Lin‐Zhu Zhang, Chao Yu, Juan Qin, Liang‐Zhu Feng, Zhuang Liu, Gao‐Jun Teng

**Affiliations:** ^1^ Center of Interventional Radiology and Vascular Surgery Nurturing Center of Jiangsu Province for State Laboratory of AI Imaging & Interventional Radiology (Southeast University) Department of Radiology Zhongda Hospital Medical School Southeast University 87 Dingjiaqiao Road Nanjing 210009 China; ^2^ National Innovation Platform for Integration of Medical Engineering Education (NMEE) (Southeast University) Nanjing 210009 China; ^3^ Basic Medicine Research and Innovation Center of Ministry of Education Zhongda Hospital Southeast University Nanjing 210009 China; ^4^ State Key Laboratory of Digital Medical Engineering Southeast University Nanjing 210009 China; ^5^ Department of Interventional Radiology The First Affiliated Hospital of Soochow University Suzhou 215006 China; ^6^ Institute of Functional Nano & Soft Materials (FUNSOM) Jiangsu Key Laboratory for Carbon‐Based Functional Materials & Devices Soochow University Suzhou 215123 China

**Keywords:** CaP nanoparticles, metabolic modulation, pickering emulsion, transarterial embolization‐immunotherapy, tumor microenvironment

## Abstract

Transarterial chemoembolization (TACE) continues to stand as a primary option for treating unresectable hepatocellular carcinoma (HCC). However, the increased tumor hypoxia and acidification will lead to the immunosuppressive tumor microenvironment (TME) featuring exhausted T cells, limiting the effectiveness of subsequent therapies following TACE. Herein, a stable water‐in‐oil lipiodol Pickering emulsion by employing calcium phosphate nanoparticles (CaP NPs) as stabilizers is developed and used to encapsulate L‐arginine (L‐Arg), which is known for its ability to modulate T‐cell metabolism. The obtained L‐Arg‐loaded CaP‐stabilized lipiodol Pickering emulsion (L‐Arg@CaPL) with great emulsion stability can not only neutralize the tumor acidity via reaction of CaP NPs with protons but also enable the release of L‐Arg, thereby synergistically promoting the reinvigoration of exhausted CD8^+^ T cells and effectively reversing tumor immunosuppression. As a result, TACE therapy with L‐Arg@CaPL shows greatly improved therapeutic responses as demonstrated in an orthotopic liver tumor model in rats. This study highlights an effective yet simple nanoparticle‐stabilized Pickering emulsion strategy to promote TACE therapy via modulation of the immunosuppressive TME, presenting great potential for clinical translation.

## Graphical abstract

L‐Arg‐loaded CaP‐stabilized lipiodol (L‐Arg@CaPL) Pickering emulsions are formulated for potentiating TACE‐immunotherapy. L‐Arg@CaPL emulsions not only neutralizes the tumor acidity via the reaction of CaP NPs with protons but also facilitates the release of L‐Arg, thereby synergistically promoting the reinvigoration of exhausted CD8^+^ T cells and effectively reversing tumor immunosuppression to promote TACE therapy.

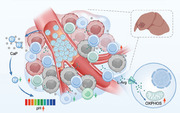

## 1 Introduction

Hepatocellular carcinoma (HCC) ranks among the foremost contributor to global cancer‐related mortality.^[^ ^1^, ^2^ ^]^ Current conventional therapies encompass surgical resection, liver transplantation, and locoregional interventions such as ablation and embolization treatments.^[^ ^3^, ^4^, ^5^ ^]^ Due to the difficulty in early diagnosis, a significant proportion of individuals with HCC receive their diagnosis at an intermediate to advanced stage, thereby missing the possibility of undergoing surgical resection or ablation.^[^ ^6^ ^]^ Transarterial embolization (TAE) or transarterial chemoembolization (TACE) has been widely applied in clinics as a minimally invasive interventional therapeutic strategy.^[^ ^7^, ^8^, ^9^, ^10^ ^]^ In this approach, embolic agents with or without chemotherapy drugs are injected into the tumor‐feeding arteries through a catheter, blocking the oxygen and nutrient supply to limit tumor growth and progression.^[^ ^11^, ^12^, ^13^ ^]^ However, TAE/TACE, as a palliative treatment, is difficult to completely inhibit tumor growth, especially for patients with late‐stage HCC.^[^ ^14^ ^]^ In recent years, systemic immunotherapies after TACE have been extensively applied to treat late‐stage HCC in clinics.^[^ ^15^, ^16^, ^17^ ^]^ Unfortunately, the response rate of such treatment strategies is still far from satisfactory, largely due to the presence of an immunosuppressive tumor microenvironment (TME) that would limit the functions of effector immune cells via different mechanisms.^[^ ^18^, ^19^ ^]^

It is well known that the TME within solid tumors is marked by numerous immunosuppressive elements, among which mild acidity and a shortage of L‐arginine (L‐Arg) are prevalent traits.^[^ ^20^, ^21^, ^22^, ^23^, ^24^ ^]^ On the one hand, tumor cells undergoing aerobic glycolysis would produce large amounts of lactic acid, which has a limiting effect on lymphocytes, leading to inhibition of their activity and proliferation.^[^ ^25^, ^26^ ^]^ In addition, this acidic TME also influences natural killer (NK) cells and macrophages, thus aggravating immunosuppressive TME.^[^ ^27^ ^]^ On the other hand, L‐Arg deficiency is another significant feature of TME since L‐Arg consumption in tumor tissues is much greater than intake.^[^ ^28^, ^29^ ^]^ Therefore, the available level of L‐Arg in TME stands as a pivotal factor influencing the cytotoxic activity of immune effector cells, including T cells.^[^ ^30^ ^]^ Increasing L‐Arg content within TME regulates metabolism, including facilitating the shift of T cells from glycolysis to oxidative phosphorylation and the production of central memory‐like cells with higher survivability, thus greatly enhancing anti‐tumor ability.^[^ ^31^ ^]^ However, it is still a challenge to deliver large amounts of L‐Arg into tumors to achieve sustained release by clinical routine means, while continuously neutralizing the intratumoral pH.

**Scheme 1:**
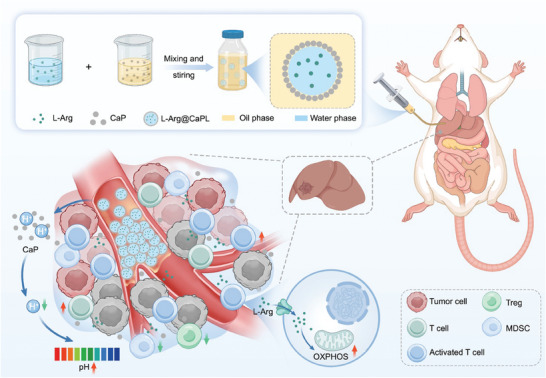
Schematic illustration of the preparation of L‐Arg‐loaded CaP‐stabilized lipiodol Pickering emulsions for potentiating TACE‐immunotherapy via neutralization of the tumor acidity and modulation of T‐cell metabolism.

Therefore, there is an urgent need to develop innovative strategies that can efficiently regulate immunosuppressive TME characterized by weak acidity and L‐Arg deficiency to reinvigorate exhausted CD8^+^ T cells, and thus reverse immunosuppressive TME to enhance the therapeutic effect of TACE. In this work, we thus designed a calcium phosphate nanoparticle (CaP NP)‐stabilized and L‐Arg encapsulated lipiodol Pickering emulsion for efficient TACE therapy of HCC tumors. In conventional TACE (cTACE) therapy, lipiodol as an embolic agent can be formulated with chemotherapeutics into lipiodol emulsions before transarterial administration, allowing efficient drug delivery directly into tumors. Herein, to enhance the stability of lipiodol emulsion and render it pH neutralization effect, CaP NPs were employed for the formulation of a stable water‐in‐oil lipiodol Pickering emulsion, in which L‐Arg was encapsulated during the emulsion preparation process (**Scheme** **1**). The resulting CaP‐stabilized lipiodol Pickering emulsion loaded with L‐Arg (L‐Arg@CaPL) demonstrated significantly enhanced emulsion stability compared to traditional lipiodol emulsion. Interestingly, such L‐Arg@CaPL could neutralize the acidic TME of tumors through the reaction of CaP NPs with protons and continuously release L‐Arg. As a result, the metabolism of T cells in the tumor would be modulated, allowing the infiltration and reinvigoration of immune‐promoting cells including CD8^+^ T cells and NK cells. Simultaneously, there was a down‐regulation in the proportion of immunosuppressive cells such as regulatory T cells (Tregs) and myeloid‐derived suppressor cells (MDSCs), indicating a shift from an immunosuppressive TME to an immune‐supportive one. Therefore, the proliferation of subcutaneous H22 mouse HCC was significantly inhibited after intratumoral injection of L‐Arg@CaPL. Moreover, L‐Arg@CaPL, when administered via intra‐arterial embolization in the tumor region, demonstrated a notable capacity to suppress the progression of orthotopic N1S1 HCC in rats, yielding significantly enhanced therapeutic efficacy in comparison to sole TAE therapy. Therefore, this study highlights an effective strategy to prepare stable embolic emulsion with pH‐regulation and T‐cell metabolic modulation capability to potentiate TACE for HCC, holding great prospects for clinical application.

## 2 Results and Discussion

### 2.1 Preparation and Characterization of L‐Arg@CaPL

**Figure 1:**
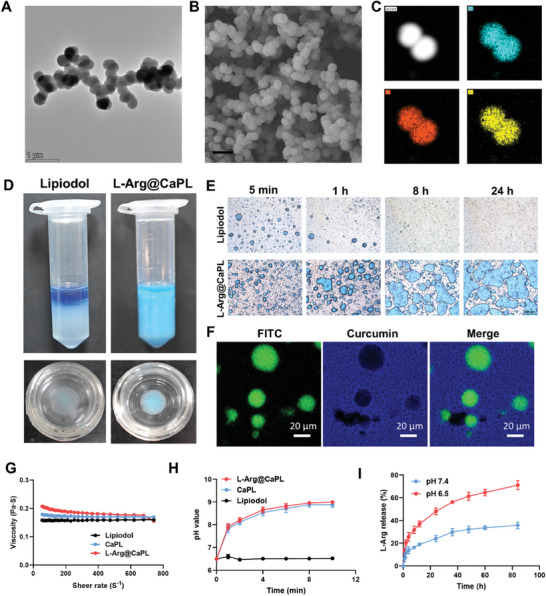
Preparation and characterization of L‐Arg@CaPL. (A) TEM image of as‐synthesized CaP NPs. (B&C) SEM image (B) and elemental mapping images (C) of Ca, P, and O within CaP NPs. Scale bar in B was 1 µm. (D) Representative optical images of conventional lipiodol emulsion, and L‐Arg@CaPL at 24 h post preparation (top) and corresponding emulsions added to water (bottom). (E) Time‐dependent optical microscopic images of conventional lipiodol emulsion, and L‐Arg@CaPL as indicated. (F) Confocal microscopic images of L‐Arg@CaPL, L‐Arg, and lipiodol labeled with FITC and curcumin. (G) The viscosity of Lipiodol, CaPL, and L‐Arg@CaPL recorded under varying vs shear rates as indicated. (H) The pH value changes of the buffer from acidity to neutrality after L‐Arg@CaPL addition via pH microelectrode. (I) Time‐dependent release profiles of L‐Arg from L‐Arg@CaPL incubated in PBS at pH 7.4 or 6.5. n = 3 biologically independent samples in (H, I). Data are presented as mean values ± SD.

First, the CaP NPs were obtained in the reverse microemulsion system.^[^ ^32^ ^]^ The transmission electron microscopy (TEM) and scanning electron microscopy (SEM) images showed that the synthesized CaP NPs had uniform spherical morphologies (**Figure** **1**A,B). Based on the dynamic light scattering (DLS) measurements, the average size of the as‐synthesized CaP NPs was ≈220 nm (Figure S1, Supporting Information). The X‐ray diffraction (XRD) results showed that the prepared CaP NPs were amorphous (Figure S2, Supporting Information), which was conducive to decomposition under acidic pH. The energy dispersive spectrometer (EDS) elemental mapping displayed a homogeneous distribution of Ca, P, and O elements in CaP NPs (Figure 1C). In addition, the chemical composition was determined by X‐ray photoelectron spectroscopy (XPS), which revealed the presence of Ca, P, and O in the sample (Figure S3, Supporting Information). Moreover, hemolysis assays of red blood cells after incubation with CaP NPs were performed to assess the biocompatibility (Figure S4, Supporting Information). The results showed that CaP NPs exhibited little hemolysis (≈3%) even at the maximum concentration of 200 µg mL^−1^, lower than the allowable hemolysis levels.

To construct lipiodol Pickering emulsion, the optimum parameters for preparing stable water‐in‐oil emulsion were first screened. Similar to the clinical observation, the water‐in‐oil emulsion formed by simply stirring the mixture of lipiodol and methylene blue (MB, color indicator) aqueous solution with a volume ratio of 2:1 exhibited inadequate stability, as shown by the rapid phase separation within 1 h (Figure 1D,E; Figure S5, Supporting Information). Under identical preparatory conditions, the addition of CaP NPs could also generate a highly stable water‐in‐oil Pickering emulsion, whose stability would be greatly improved with the addition of CaP NPs in a dose‐dependent manner (Figures S6–S8, Supporting Information). In addition, the concurrent incorporation of CaP and L‐Arg could form water‐in‐oil emulsion of L‐Arg@CaPL with further improved stability (Figure S9, Supporting Information). These findings collectively illustrated that the adsorption of unbound fatty acids in lipiodol onto CaP NPs would enhance the surface hydrophobicity of CaP NPs, thereby facilitating the formation of water‐in‐oil CaP‐stabilized Pickering emulsions.^[^ ^33^ ^]^ Next, we characterized the obtained CaNP‐stabilized Pickering emulsion. By monitoring the fluorescence of fluorescein isothiocyanate (FITC, serving as a surrogate for L‐Arg), it was found that the small molecule FITC would reside within aqueous microdroplets encircled by hydrophobic lipiodol labeled with blue fluorescent curcumin (Figure 1F).^[^ ^33^ ^]^ Then, the viscosity of these lipiodol emulsions was assessed using a rotary rheometer to determine their availability for injection into the hepatic arteries. L‐Arg@CaPL demonstrated a viscosity of ≈0.2 Pa·S at a shear rate of 300 s^−1^, which was consistent with the injection rate of 5–10 mL min^−1^ during clinical TAE surgery (Figure 1G), affirming its injectability adequacy for TAE therapy. Since CaP NPs could act as effective proton sponges to neutralize acidity, the acidic buffer showed a rapid increase in pH after the addition of L‐Arg@CaPL or CaPL (Figure 1H). Moreover, it was demonstrated that L‐Arg@CaPL, incubated under acidic conditions with pH ≈6.5, showed accelerated release kinetics of L‐Arg in contrast to those incubated under physiological pH ≈7.4 (Figure 1I), demonstrating that L‐Arg@CaPL could achieve efficient pH‐responsive payload release.

### 2.2 Intratumoral Retention of L‐Arg@CaPL to Allow TME Regulation

**Figure 2:**
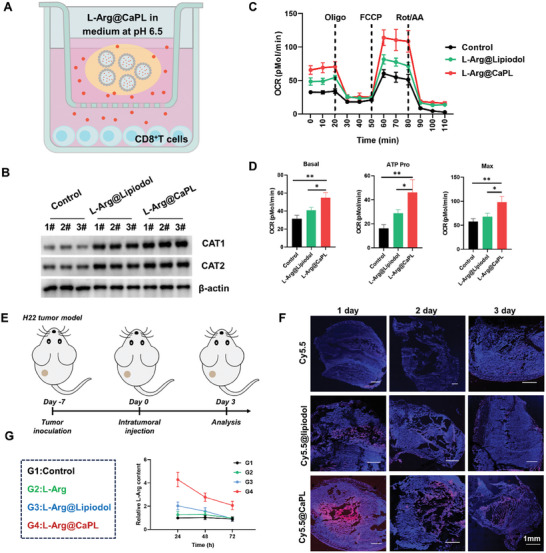
Intratumoral retention and immunosuppressive TME regulation of L‐Arg@CaPL. (A) A schematic diagram of L‐Arg@CaPL regulating T‐cell metabolic function in vitro. (B) The expression levels of CAT1 and CAT2 in CD8^+^ T cells detected via western blotting analysis. (C) Analyzes of oxygen consumption rate (OCR) of T cells after different treatments via Seahorse extracellular experiment. (D) OCR quantifications of basal respiration, ATP production, and maximal respiration. (E) A schematic diagram of experimental schedule. (F) Confocal images of tumor slices collected from H22 tumor‐bearing mice with intratumoral injection of free Cy5.5, Cy5.5@lipiodol, and Cy5.5@CaPL at the indicated time points. (G) Time‐dependent relative L‐Arg content within H22 tumors after different treatments measured via ELISA as indicated. n = 3 biologically independent animals. Data are presented as mean values ± SD. One‐way analysis of variance (ANOVA) was used for multiple comparisons. *P < 0.05, **P < 0.01, and ***P < 0.001.

The remarkable acidity neutralization of L‐Arg@CaPL and the sustained release capability of L‐Arg prompted further assessment of the ability of L‐Arg@CaPL to regulate T cell metabolic function in vitro (**Figure** **2**A). First, the expression levels of cationic amino acid transporters 1(CAT1) and CAT2 in T cells were assessed using western blotting analysis (Figure 2B). The results revealed a significant increase in both CAT1 and CAT2 following treatment with L‐Arg@CaPL, indicating enhanced uptake of L‐Arg. To determine the ability of L‐Arg@CaPL to regulate T‐cell metabolism, the Seahorse extracellular experiment was used for extracellular flux analysis (Figure 2C). The results revealed higher basal respiration, ATP production, and maximal oxygen consumption rates (OCR) of T cells treated with L‐Arg compared to control, indicating that L‐Arg‐treated T cells were more reliant on oxidative phosphorylation (Figure 2C,D). In addition, L‐Arg@CaPL‐treated T cells showed the highest basal respiration, ATP production, and maximal OCR, suggesting that T cells treated with L‐Arg@CaPL had a greater metabolic reserve to utilize mitochondrial respiration. L‐Arg@CaPL treatment also resulted in a significant enhancement of the mitochondrial spare respiratory capacity (SRC) in T cells (Figure S10, Supporting Information). Moreover, extracellular acidification rate (ECAR) was further performed to substantiate the metabolic shift of T cells from glycolysis to oxidative phosphorylation (Figure S11, Supporting Information). The results demonstrated that the administration of L‐Arg@CaPL exhibited a significant reduction in both the basal and maximal glycolytic capacity. Furthermore, the frequencies of central memory T cells (T_CM,_ CD44^+^CD62L^+^) and effector memory T cells (T_EM,_ CD44^+^CD62L^−^) were significantly augmented following treatment with L‐Arg@CaPL (Figure S12, Supporting Information). These results suggested that L‐Arg supplementation promoted the transformation of T cells from glycolysis to oxidative phosphorylation, which was conducive to the generation of central memory‐like cells with higher survival ability, and thus greatly enhanced the anti‐tumor ability of those T cells.^[^ ^20^, ^30^ ^]^

Moreover, in light of the enhanced stability of CaNP‐stabilized lipiodol Pickering emulsion, we proceeded to assess its ability to facilitate the intratumoral retention of its payload molecules such as Cy5.5, in mice bearing subcutaneous H22 tumors (Figure 2E). By detecting Cy5.5 fluorescence in tumor sections by confocal microscopy, it could be seen that Cy5.5@CaPL injected into the tumor showed prolonged retention of Cy5.5 signals for more than 3 days (Figure 2F), in marked contrast to tumors with intratumoral injection of Cy5.5@lipiodol or Cy5.5 that showed rather rapid clearance of Cy5.5 signals. The superior capacity of CaPL in facilitating the enduring preservation of Cy5.5‐BSA was additionally validated through the documentation of Cy5.5 fluorescence using in vivo fluorescence imaging (Figure S13, Supporting Information). Moreover, through quantitative statistical analysis via enzyme‐linked immunosorbent assay (ELISA), it could be seen that the L‐Arg content retained in the tumor of the L‐Arg@CaPL group post intratumoral injection was much higher compared to that in tumors injected with free L‐Arg or L‐Arg@Lipiodol (Figure 2G). Collectively, these results proved that CaNP‐stabilized lipiodol Pickering emulsion facilitated sustained retention of its payloads, such as L‐Arginine, within tumors, owing to its exceptional stability.

### 2.3 In Vivo Therapeutic Potency of L‐Arg@CaPL in H22 Tumor Model

**Figure 3:**
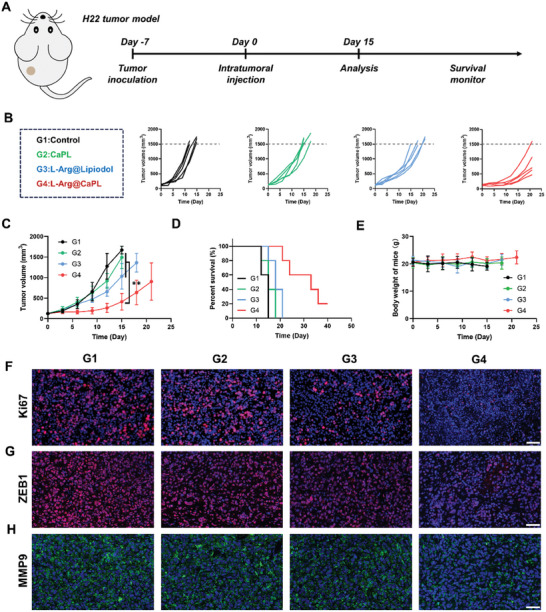
I*n vivo* therapeutic potency of L‐Arg@CaPL in H22 tumor model. (A) A schematic diagram of the therapeutic schedule. (B‐E) Individual tumor growth curves (B), average tumor sizes (C), survival rate (D), and average body weights (E) of H22 tumor‐bearing mice after receiving various treatments as indicated (Control, CaPL, L‐Arg@Lipiodol, and L‐Arg@CaPL). (F‐H) Ki67, ZEB1, and MMP9 staining of tumor slices collected from the treated mice at 15 day post various treatments. Scale bar: 50 µm. n = 5 biologically independent animals. Data are presented as mean values ± SD. One‐way analysis of variance (ANOVA) was used for multiple comparisons. **P < 0.01.

Then, four other groups of mice with H22 tumors subsequently underwent the following interventions including control, CaPL, L‐Arg@Lipiodol, and L‐Arg@CaPL, aimed at evaluating the therapeutic efficacy of L‐Arg@CaPL (**Figure** **3**A). Remarkably, the tumor growth of the L‐Arg@CaPL group was markedly suppressed, whereas CaPL or L‐Arg@lipiodol treatment exhibited only a slight inhibitory effect (Figure 3B–D). Notably, administration of L‐Arg@CaPL could significantly prolong animal survival time. In addition, the mice subjected to different treatments exhibited no significant decrease in body weight throughout the observation period, suggesting the outstanding biocompatibility of our proposed emulsions at the tested dosage (Figure 3E). Moreover, to further validate the anti‐tumor performance, various histological analyses, such as Ki67 staining, zinc‐finger‐enhancer binding protein 1 (ZEB1) staining, and matrix metalloproteinase 9 (MMP9) staining, were conducted (Figure 3F–H). After administration of L‐Arg@CaPL, the expressions of typical markers of cell proliferation and cell metastasis were significantly decreased, suggesting that L‐Arg@CaPL had an inhibitory effect on cell proliferation and metastasis. Meanwhile, the pH‐sensitive fluorescent staining of BCECF‐AM was performed on tumor sections after different treatments to assess the acidity level of the tumors. The fluorescence signals of groups 2 and 4 were significantly enhanced, indicating that emulsions containing CaP NPs could significantly neutralize acidic TME (Figure S14, Supporting Information). Collectively, these results evinced that L‐Arg@CaPL efficaciously inhibited the growth of H22 tumors without notable adverse reactions.

### 2.4 L‐Arg@CaPL‐Mediated Reinvigoration of Exhausted CD8^+^ T Cells and Reversal of Immunosuppressive TME

**Figure 4:**
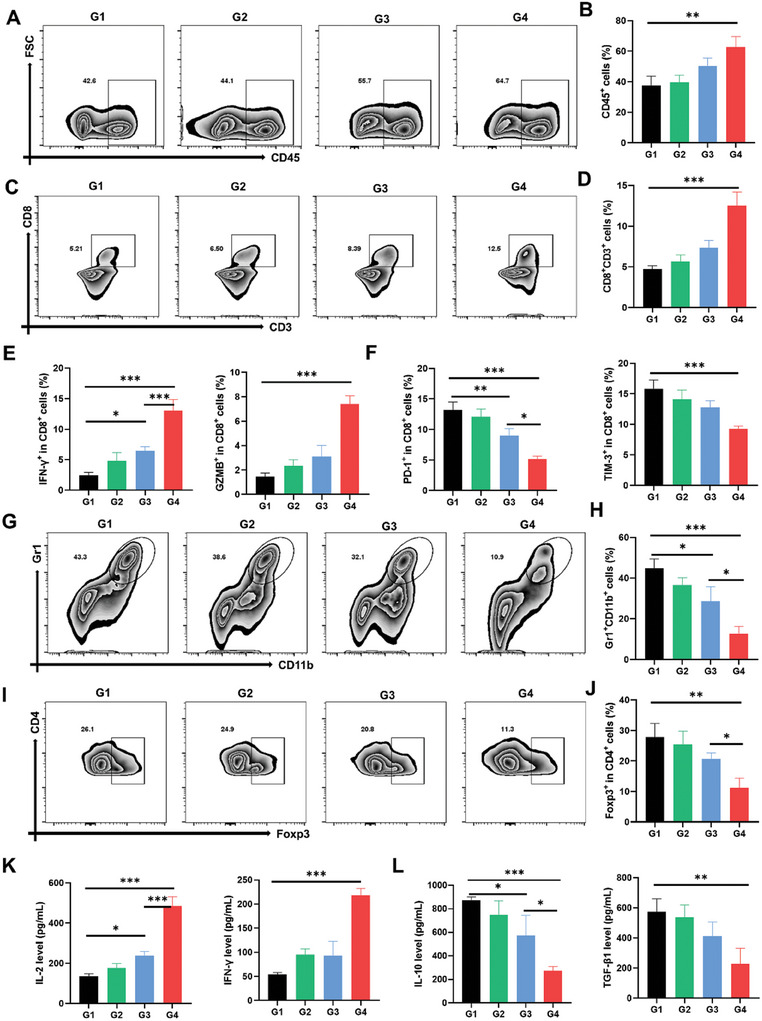
L‐Arg@CaPL‐mediated reinvigoration of exhausted CD8^+^ T cells and reversion of immunosuppressive TME in H22 tumor model. (A‐B) Representative flow cytometric analysis and quantification results of the frequencies of CD45^+^ T cells. (C‐D) Representative flow cytometric analysis and quantification results of the frequencies of CD3^+^CD8^+^ T cells. (E) Quantification results of the frequencies of effector CD8^+^ T cells (IFN‐γ^+^CD8^+^, GZMB^+^CD8^+^). (F) Quantification results of the frequencies of exhausted CD8^+^ T cells (PD‐1^+^CD8^+^, TIM‐3^+^CD8^+^). (G‐H) Representative flow cytometric analysis and quantification results of the frequencies of MDSCs. (I‐J) Representative flow cytometric analysis and quantification results of the frequencies of Treg cells. (K‐L) Secretion levels of IL‐2, IFN‐γ, IL‐10, and TGF‐β1 in the tumors of mice post varying treatments as indicated. n = 3 biologically independent animals. G1–G4 represent control; CaPL; L‐Arg‐Lipiodol; L‐Arg@CaPL. Data are presented as mean values ± SD. One‐way analysis of variance (ANOVA) was used for multiple comparisons. *P < 0.05, **P < 0.01, and ***P < 0.001.

After confirming the capability of L‐Arg@CaPL to neutralize tumor acidity and promote L‐Arg retention, the ability of L‐Arg@CaPL to reinvigorate exhausted CD8^+^ T cells and reverse immunosuppressive TME was further carefully evaluated. H22 tumor tissues from mice were homogenized and stained with fluorescent antibodies for flow cytometry on day 7 after intratumoral injection of L‐Arg@CaPL. Compared with the control group, the intracellular proportions of CD45^+^ leukocyte cells and CD3^+^CD8^+^ T cells in the CaPL group were upregulated due to tumor acidity neutralization (**Figure** **4**A–D), which reversed acidic TME that inhibited T cell activation and proliferation.^[^ ^15^, ^34^ ^]^ In addition, a similar trend was obtained in the L‐Arg@Lipiodol group, indicating that the release of L‐Arg promoted the regulation of T‐cell metabolism.^[^ ^30^, ^31^ ^]^ Notably, L‐Arg@CaPL treatment resulted in a notable augmentation of intracellular CD45^+^ leukocyte cells cytotoxic and CD3^+^CD8^+^ T cells due to the simultaneous consumption of protons in TME and the regulation of T‐cell metabolism. The ATP contents in CD8^+^ T cells within tumors were evaluated to determine the variations resulting from the regulation of T‐cell metabolism, as oxidative phosphorylation is primarily responsible for ATP production (Figure S15, Supporting Information). The findings demonstrated a significant upregulation in ATP contents of CD8^+^ T cells following treatment with L‐Arg@CaPL, indicating an enhancement in oxidative phosphorylation. Significantly, such treatment was also able to promote the expression of effector cytokine IFN‐γ and cytolytic granzyme B (GZMB) inside CD3^+^CD8^+^ T cells (Figure 4E; Figure S16, Supporting Information). In addition, it was found that treatment of L‐Arg@CaPL could lead to reduced expressions of programmed cell death protein‐1 (PD‐1), T‐cell immunoglobulin and mucin‐domain containing‐3 (TIM‐3), which are recognized markers of T‐cell exhaustion observed on CD3^+^CD8^+^ T cell surfaces (Figure 4F). Moreover, the proportion of Ki67^+^CD8^+^ T cells was significantly increased following L‐Arg@CaPL treatment (Figure S17, Supporting Information). Meanwhile, the immunofluorescence staining images of H22 tumors revealed an increased proportion of CD25^+^CD8^+^ T cells (Figure S18, Supporting Information). Furthermore, the frequencies of T_CM_ (CD44^+^CD62L^+^) and T_EM_ (CD44^+^CD62L^−^) were significantly augmented following treatment with L‐Arg@CaPL (Figure S19, Supporting Information). These findings collectively affirmed that simple L‐Arg@CaPL treatment effectively revitalized fatigued CD8^+^ T cells and enhanced their cytolytic functions by simultaneously regulating acidic TME and regulating T‐cell metabolism.^[^ ^35^ ^]^

Moreover, we further found that treatment with L‐Arg@CaPL could also result in an obvious reduction of intratumoral abundances of immunosuppressive cells including MDSCs (CD45^+^CD11b^+^Gr‐1^+^, Figure 4G,H) and Tregs (CD3^+^CD4^+^Foxp3^+^, Figure 4I,J). However, regulation of acidic TME alone through CaPL or regulation of T cell metabolism through L‐Arg had a relatively weak effect on reducing the proportion of immunosuppressive cells such as MDSCs and Tregs. Furthermore, increased secretion of IL‐2, IFN‐γ, TNF‐α, and reduced secretion of IL‐10, and TGF‐β1 were further observed in the supernatants of these tumors following excision from the mice treated with L‐Arg@CaPL (Figure 4K,L; Figure S20, Supporting Information). Taken together, our results demonstrated that administration of L‐Arg@CaPL could effectively modulate the immunosuppressive TME by simultaneously neutralizing the acidic TME and regulating T‐cell metabolism, thus strongly activating anti‐tumor immunity.

### 2.5 In Vivo TACE Treatment with L‐Arg@CaPL in an Orthotopic N1S1 Rat Tumor Model

**Figure 5:**
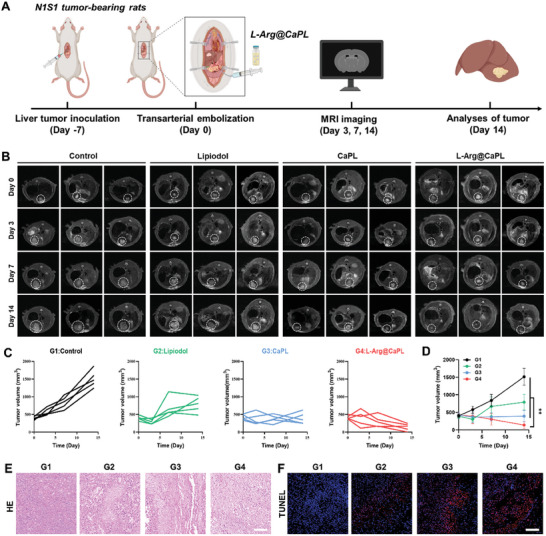
In vivo TACE treatment with L‐Arg@CaPL in an orthotopic N1S1 rat tumor model. (A) A schematic diagram of the TACE treatment schedule in N1S1 bearing rats. (B) Representative T2 contrast‐enhanced MR scanning of N1S1‐bearing rats with different treatments as indicated. (C‐D) Individual (C) and average tumor growth curves (D) of different groups of N1S1 tumor‐bearing rats after various treatments as indicated. (E‐F) H&E and TUNEL staining of tumor slices collected from these N1S1 tumor‐bearing rats with various treatments as indicated. n = 5 biologically independent animals. Scale bar: 100 µm. Data are presented as mean values ± SD. One‐way analysis of variance (ANOVA) was used for multiple comparisons. **P < 0.01.

TACE, serving as a minimally invasive therapeutic modality, has garnered extensive utilization in the clinical treatment of advanced HCC.^[^ ^10^, ^36^ ^]^ Inspired by the excellent stability and antitumor ability of L‐Arg@CaPL, our investigation extended to exploring its inhibitory potential against orthotopic N1S1 tumors. After the successful establishment of the tumor model by injecting N1S1 cells into the left lobe of the liver for 7 days, the rats with orthotopic N1S1 tumors were randomly assigned into different groups (n = 5). A 3.0‐T magnetic resonance (MR) imaging system was used to monitor changes in tumor volume with intraperitoneal injection of commercial gadolinium contrast agents on day 0 before treatments and days 3, 7, and 14 after various treatments (**Figure** **5**A,B). The N1S1 tumor without any treatment grew rapidly from an initial volume of ≈450 to ≈1500 mm^3^ within two weeks (Figure 5C,D). In comparison with tumors in untreated rats, the N1S1 tumors in rats after TAE treatment with lipiodol exhibited partially delayed growth, with a final volume of ≈800 mm^3^. In addition, CaPL treatment further enhanced the inhibition of N1S1 tumor growth, with a final volume of ≈450 mm^3^. Remarkably, treatment with L‐Arg@CaPL exhibited exceptional efficacy in suppressing tumors, with only small lesion tissues presented on MR imaging on day 14 post the corresponding treatment. Moreover, to provide additional validation for the therapeutic outcomes following TACE therapy, histopathological examinations by hematoxylin and eosin (H&E) and TdT‐mediated dUTP nick‐end labeling (TUNEL) staining were carried out to perform the tumor damage statuses of the tumor slices obtained from variously treated rats (Figure 5E,F). Compared with those in the control groups, tumors treated with L‐Arg@CaPL exhibited the most severe nuclear shrinkage and fragmentation. The fluorescence signal of TUNEL, a marker of apoptosis, was significantly increased, suggesting that L‐Arg@CaPL could significantly induce tumor cell death. To further elucidate the efficacy of combining L‐Arg@CaPL with TACE, we conducted a comparative analysis between this combination therapy and direct intratumoral injection (Figure S21, Supporting Information). The results demonstrated a significantly superior effectiveness of the combination treatment over intratumoral injection, thereby providing additional evidence for the potential enhancement of TACE through our developed strategy.

To evaluate the safety and biocompatibility of our proposed strategies, healthy rats after TACE with L‐Arg@CaPL were sacrificed at 30 days and the major organs and blood samples were collected. The dosage of L‐Arg, CaP, and Lipiodol were 0.6 mg, 1.5 mg, and 150 µL, respectively. No significant damage or inflammation was detected in the vital organs of the rats, indicating that the L‐Arg@CaPL‐enhanced TACE strategy had good biocompatibility (Figure S22, Supporting Information). In addition, the main indicators of blood biochemistry were further evaluated as to whether such a therapeutic strategy would result in potentially toxic side effects. Total bilirubin (TBIL), serum calcium (Ca^2+^), alanine aminotransferase (ALT), and serum creatinine (CREA), were within the reference range similar to the control group, indicating that L‐Arg@CaPL treatment caused no notable toxicity (Figure S23, Supporting Information). Taken together, these results demonstrated that L‐Arg@CaPL could effectively inhibit the progression of N1S1 tumors without evident adverse reactions.

### 2.6 L‐Arg@CaPL‐Mediated Reversion of Tumor Immunosuppression in an Orthotopic N1S1 Rat Tumor Model

**Figure 6:**
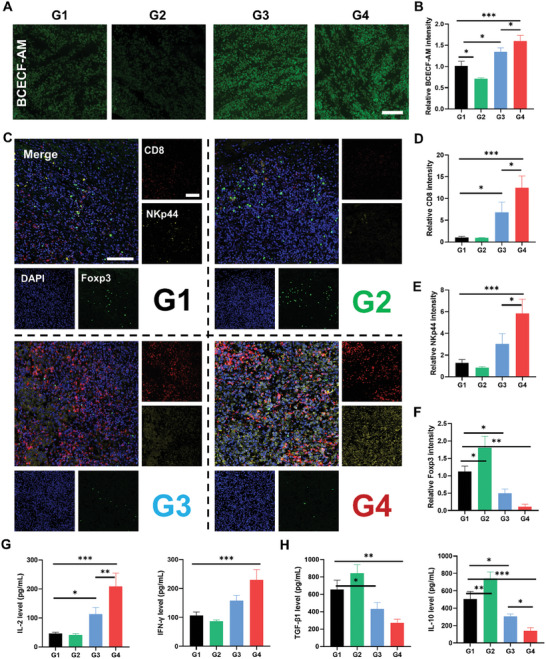
L‐Arg@CaPL‐mediated reversion of tumor immunosuppression in an orthotopic N1S1 rat tumor model. (A‐B) Confocal images and semi‐quantitative analysis of tumor slices after BCECF‐AM staining collected from N1S1 tumor‐bearing rats post various treatments as indicated. Scale bar: 100 µm. (C) Immunofluorescent‐stained images showing the infiltration of CD8^+^ T cells, NK cells, and Treg cells within N1S1 tumors. Scale bar: 100 µm. (D‐F) Quantitative analysis of signal intensities of CD8^+^ T cells, NK cells, and Treg cells based on stained slices. (G‐H) Secretion levels of IL‐2, IFN‐γ, TGF‐β1, and IL‐10 in the tumors of rats post varying treatments as indicated. n = 3 biologically independent animals. Data are presented as mean values ± SD. One‐way analysis of variance (ANOVA) was used for multiple comparisons. *P < 0.05, **P < 0.01, and ***P < 0.001.

Furthermore, another four groups of rats carrying N1S1 orthotopic HCCs were established and subjected to identical treatments as previously described to explore the ability of L‐Arg@CaPL to regulate immunosuppressive TME. First, BCECF‐AM staining was performed on tumor sections after different treatments to evaluate tumor acidity. The fluorescence signals of groups 3 and 4 were significantly enhanced, indicating that emulsions containing CaP NPs could significantly neutralize acidic TME (**Figure** **6**A,B). Immunofluorescence‐stained images of N1S1 tumors were acquired subsequently to comprehend the mechanism underlying the antitumor effect induced by L‐Arg@CaPL‐mediated TACE (Figure 6C–F). Interestingly, the infiltration of immune‐promoting cells including CD8^+^ T cells and NK cells in the L‐Arg@CaPL treatment group was significantly increased compared with other groups, thus strongly facilitating the cell‐killing effect. Tumors in the L‐Arg@CaPL group showed reduced proportion of Treg cells, a class of immunosuppressive cells that promote tumor progression by suppressing anti‐tumor immunity. Additionally, treatment with L‐Arg@CaPL resulted in a significant increase in ATP content within CD8^+^ T cells present in tumors, indicating an augmentation of oxidative phosphorylation (Figure S24, Supporting Information). Furthermore, the frequency of T_CM_ exhibited a significant increase following treatment with L‐Arg@CaPL (Figure S25, Supporting Information). To further validate the reversal of the immunosuppressive TME, the pro‐inflammatory and anti‐inflammatory cytokine levels within tumor tissues were assessed (Figure 6G,H; Figure S26, Supporting Information). In the L‐Arg@CaPL group, there was an increase in the expression of pro‐inflammatory cytokines, including IL‐2, IFN‐γ, and TNF‐α, and a decrease in the production of anti‐inflammatory cytokines, including TGF‐β1, and IL‐10. Taken together, our results suggested that administration of L‐Arg@CaPL could effectively activate exhausted T cells, recruit NK cells for effective tumor invasion, and reduce the abundance of immunosuppressive immune cells, thereby efficiently boosting anti‐tumor immunity.

## 3 Conclusion

In this study, a CaP‐stabilized and L‐Arg encapsulated lipiodol Pickering emulsion with TME regulating capability was concisely prepared for efficient TACE therapy of HCC tumors. The yielded L‐Arg@CaPL showed greatly improved emulsion stability over conventional lipiodol emulsion, and displayed pH‐triggered release of encapsulated L‐Arg facilitated by the degradation of CaP NPs within the acidic TME. Interestingly, L‐Arg@CaPL exhibited a remarkable capability to neutralize the acidic TME through proton interaction, and subsequently modulate T‐cell metabolism via continuous L‐Arg release. This in turn promoted the revitalization of exhausted CD8^+^ T cells, thereby enhancing anti‐tumor immunity. Consequently, intratumoral administration of L‐Arg@CaPL significantly impeded the growth of subcutaneous H22 mouse HCC by activating the immune system. More significantly, L‐Arg@CaPL, when intra‐arterially embolized in the tumor region, demonstrated effective suppression of orthotopic N1S1 HCC growth in rats, yielding markedly enhanced therapeutic efficacy compared to TAE therapy alone. Therefore, this study underscores an efficient approach in fabricating stable embolic emulsion with pH regulation and T‐cell metabolic modulation properties to potentiate TACE therapy for HCC, by regulating the immunosuppressive TME.

On the basis of the published real‐world data and ongoing randomized controlled trials (RCTs), combining TACE with immunotherapy‐based treatment strategies has demonstrated positive results for the management of middle or late‐stage HCC.^[^ ^10^, ^16^, ^37^, ^38^ ^]^ In clinical practice, such combined therapies have been strongly recommended for locally advanced HCC in China. However, the TACE‐induced tumor acidification and hypoxia would lead to reduced numbers of peripheral CD4^+^ T cells and infiltrating CD8^+^ T cells, while augmenting the recruitment of regulatory T cells and myeloid‐derived suppressor cells, resulting in an immunosuppressive TME.^[^ ^39^, ^40^ ^]^ Our L‐Arg@CaPL emulsion could not only neutralize the tumor acidity but also release L‐Arg to promote the reinvigoration of exhausted CD8^+^ T cells, synergistically and effectively reverse tumor immunosuppression. Therefore, we expect that such L‐Arg@CaPL emulsion may be particularly meaningful if used for combined TACE‐immunotherapy. Given the excellent biocompatibility of all components employed in formulating these emulsions, the TACE regimen utilizing L‐Arg@CaPL exhibits significant potential for forthcoming clinical translation for the management of patients with HCC.

## 4 Experimental Section

All materials, characterization, and experimental details have been added in supporting information.

## Conflict of Interest

The authors declare no conflict of interest.

## Author Contributions

D.W., L.Z., and W.H.Y. contributed equally to this work. G.T. and Z.L. conceived and planned the study. D.W., L.Z., L.Z. and W.Y. conducted all the experiments and performed the data analysis and interpretation. C.Y. and J.Q. also participated in the data analysis and interpretation of the results. G.T., Z.L. and L.F. provided critical comments. D.W. and L.Z. wrote the manuscript, which was reviewed and edited by all coauthors.

## Supporting information



Supporting Information

## Data Availability

The data that support the findings of this study are available from the corresponding author upon reasonable request.
