# Supplementary material for: Arginine‐Loaded Nano‐Calcium‐Phosphate‐Stabilized Lipiodol Pickering Emulsions Potentiates Transarterial Embolization‐Immunotherapy
Source: Adv Sci (Weinh). 2024 Dec 16;12(6):2410484. doi: 10.1002/advs.202410484 (PMC11809372; doi:10.1002/advs.202410484)
Supplement: Supplementary file 1 — Supporting Information [file ADVS-12-2410484-s001.docx]

Supporting Information

**Arginine-Loaded Nano-Calcium-Phosphate-Stabilized Lipiodol Pickering Emulsions Potentiates Transarterial Embolization-Immunotherapy**

*Duo Wang^a,b,c,d,1^, Lei Zhang^a,b,c,d,1,*^, Wei-Hao Yang^e,1^, Lin-Zhu Zhang^a,b,c,d^, Chao Yu^a,b,c,d^, Juan Qin^a,b,c,d^, Liang-Zhu Feng ^f^, Zhuang Liu^f, *^, Gao-Jun Teng^a,b,c,d,*^*

^a^ Center of Interventional Radiology and Vascular Surgery, Nurturing Center of Jiangsu Province for State Laboratory of AI Imaging & Interventional Radiology (Southeast University), Department of Radiology, Zhongda Hospital, Medical School, Southeast University, 87 Dingjiaqiao Road, Nanjing 210009, China

^b^ National Innovation Platform for Integration of Medical Engineering Education (NMEE) (Southeast University), Nanjing 210009, China

^c^ Basic Medicine Research and Innovation Center of Ministry of Education, Zhongda Hospital, Southeast University, Nanjing 210009, China

^d^ State Key Laboratory of Digital Medical Engineering, Southeast University, Nanjing 210009, China

^e^ Department of Interventional Radiology, The First Affiliated Hospital of Soochow University, Suzhou 215006, China

^f^ Institute of Functional Nano & Soft Materials (FUNSOM), Jiangsu Key Laboratory for Carbon-Based Functional Materials & Devices, Soochow University, Suzhou 215123, China

*To whom correspondence should be addressed:

*E-mail addresses:* zhang_lei@seu.edu.cn (L. Zhang), zliu@suda.edu.cn (Z. Liu), gjteng@seu.edu.cn(G.J. Teng).

^1^ These authors contributed equally to this work.

**Experimental section**

**Materials**

CaCl_2_, Na_3_PO_4_⋅12H_2_O were purchased from Sinopharm Chemical Reagent Co., Ltd. 1, 2-dioleoyl-sn-glycero-3-phosphate (DOPA) was purchased from Avanti Lipids Polar, Inc. Lipiodol was purchased from Jiangsu Hengrui Pharmaceutical Company. Antibodies for fluorescence microscope, and flow cytometry were purchased from BioLegend.

**Preparation and characterization of** **CaP NPs**

The CaP NPs were prepared in the reverse microemulsion system. The calcium phase was prepared by adding CaCl_2_ (500 mM, pH = 9.0) into a 15 mL solution. To obtain the phosphate phase, 600 μL of 25 mM Na_3_PO_4_ was dispersed in another 15 mL solution, into which 400 μL of 20 mg mL^-1^ DOPA in chloroform was added. After intensive mixing, the calcium phase was added dropwise to the phosphate phase to prepare CaP NPs. After incubation for 12 h, 30 mL absolute ethanol was added to break the emulsion, and the product was collected by centrifugation at 10,000 g for 15 min and purified by washing with ethanol three times. The morphologies and elemental composition of CaP NPs were determined using a Tecnai F20 transmission electron microscope (TEM) and ZEISS G500 scanning electron microscope (SEM). The hydrodynamic size distribution was measured by a Malvern Zetasizer (Nano ZS90).

**Preparation and characterization of Lipiodol, CaPL, L-Arg@Lipiodol, and L-Arg@CaPL**

Lipiodol emulsions were prepared by mixing deionized water with Lipiodol at a volume ratio of 1:2 under magnetic stirring. To prepare CaPL, CaP NPs were mixed with Lipiodol under magnetic stirring. L-Arg@Lipiodol and L-Arg@CaPL were prepared via the same protocol with the addition of corresponding agents. To evaluate the stability of Lipiodol, CaPL, and L-Arg@CaPL, their phase separation profiles were monitored by using a digital camera at determined intervals. Their microscopic morphologies were obtained by using a Leica fluorescence optical microscope. Their viscosities were measured by using a rotary rheometer.

**pH-responsive release of L-Arg from L-Arg@CaPL**

To study the pH-responsive release profile of L-Arg of L-Arg@CaPL, L-Arg@CaPL was immersed in PBS at pH 6.5 and 7.4, followed by being incubated at 37 ^o^C. At designed time intervals, the supernatants were collected to measure the amount of released L-Arg.

**OCR measurements.**

Measurements were performed using extracellular flux analyzer (Seahorse Bioscience). Naive CD8^+^ T cells were sorted and activated with plate-bound CD3 and CD28 antibodies. Four days later, cells were pooled, carefully counted and plated in serum-free unbuffered RPMI-1640 medium onto Seahorse cell plates. Oligomycin, Carbonyl cyanide-4-(trifluoromethoxy)phenylhydrazone (FCCP) and rotenone/antimycin A (Rot/AA) were injected.

**Western blotting.**

The CD8^+^ T cells were collected in 4 ^o^C cell lysis buffer for Western blotting analysis. The protein concentration was calculated with a bicinchoninic acid (BCA) protein test kit. SD-PAGE was used to load and separate the proteins. The proteins were then transferred to polyvinylidene difluoride membrane in a Tris-glycine transfer buffer and blocked with 5% bovine serum albumin (BSA) for 2 h at room temperature. The membrane was incubated with primary antibodies (anti-CAT1 and anti-CAT2) at 4 ^o^C overnight. The membranes were washed and incubated with secondary HRP-conjugated antibody (anti-rabbit IgG) at 25 ^o^C for 2 h. The membranes were washed and imaged with a Gel Logic system.

**Animal experiments**

Male Balb/c mice (6-8 weeks), and male Sprague-Dawley (SD) rats (300~350 g) were purchased from Changzhou Cavins Biological Technology Co. Ltd. To construct the subcutaneous H22 tumor model, H22 cells (2 × 10^6^) dispersed in ~50 μL of PBS were subcutaneously inoculated to the back of each Balb/c mouse. To establish the orthotopic N1S1 tumor model, N1S1 cells (6 × 10^6^) dispersed in ~80 μL of PBS containing 30% Matrigel (Corning) were inoculated into the right lower liver lobe of each SD rat under anesthesia. All animal experiments have been approved by Animal Experimental Ethical Inspection Form of Southeast University with an approval number: 20230615030.

To assess the intratumoral retention behaviors of Lipiodol, L-Arg@Lipiodol, and L-Arg@CaPL, 12 mice bearing subcutaneous H22 tumors were randomly divided into three groups (n = 3) and then intratumorally injected with free Cy5.5, Cy5.5@lipiodol, and Cy5.5@CaPL at the same dose of Cy5.5, respectively. These treated mice were imaged via in vivo fluorescence imaging system to record the Cy5.5 fluorescence intensity. In addition, at 24 h, 48 h, and 72 h post-injection, one mouse in each group was randomly picked out and sacrificed with their tumors collected for CLSM observation. For determination of L-Arg content, the mice bearing subcutaneous H22 tumors were divided into four groups: Control, L-Arg, L-Arg@Lipiodol, and L-Arg@CaPL. Subsequently, the L-Arg concentration within the tumor was quantified using an ELISA kit.

To evaluate the therapeutic efficacy of L-Arg@CaPL, mice bearing subcutaneous H22 tumors (~150 mm^3^) were randomly divided into four groups and received the following intratumoral injections: G1: Control; G2: CaPL; G3: L-Arg@Lipiodol; and G4: L-Arg@CaPL. The intratumoral injection doses of Lipiodol, CaP, and L-Arg were 2.5 mL kg^-1^, 50 mg kg^-1^, and 20 mg kg^-1^, respectively. Since then, the length and width of each tumor and the body weight of each mouse were recorded every other day by using a Vernier caliper and digital balance, respectively. The tumor volumes were calculated by following the formula of tumor volume = (width^2^ × length)/2. In addition, at two days post intratumoral injection, one mouse of each group was randomly picked out and sacrificed with their tumors collected for Ki67, ZEB1, and MMP9 staining.

To evaluate the effects of L-Arg@CaPL treatment on immunosuppressive TME, these tumors in mice after different treatments were resected, and then cut into small pieces, digested with a mixture solution of collagenase I, collagenase IV, and hyaluronidase (1.5 mg mL^-1^) and filtered with a mesh filter (200 mesh) to prepare single-cell suspensions. After being stained with corresponding fluorescent antibodies according to the vendors’ protocols, these single-cell suspensions were subjected to flow cytometry for analyzing intratumoral abundances of CD3^+^ T cells, CD3^+^CD8^+^ T cells, TIM-3^+^CD3^+^CD8^+^ T cells, PD-1^+^CD3^+^CD8^+^ T cells, IFN-γ^+^CD3^+^CD8^+^ T cells, GZMB^+^CD3^+^CD8^+^ T cells, CD45^+^ white cells, CD45^+^Gr1^+^CD11b^+^ MDSC cells and CD3^+^CD4^+^Foxp3^+^ Treg cells. Additionally, the secretion levels of diverse cytokines including IL-2, IFN-γ, TNF-α, IL-10, and TGF-β1 in the supernatant of these tumor lysates were determined by using corresponding commercial ELISA kits following the vendors’ protocols. To further elucidate the mechanism of T cell metabolism enhancement therapy regulated by L-Arg@CaPL, these spleens in mice after different treatments were resected to prepare single-cell suspensions. After being stained with corresponding fluorescent antibodies according to the vendors’ protocols, these single-cell suspensions were subjected to flow cytometry for analyzing intratumoral abundances of central memory T cells (T_CM_, CD44^+^CD62L^+^), effector memory T cells (T_EM_, CD44^+^CD62L^−^).

To evaluate the TACE treatment efficacy of L-Arg@CaPL toward orthotopic N1S1 HCCs, a total of 20 rats bearing orthotopic N1S1 hepatocellular carcinoma tumors (~450 mm^3^) were randomly divided into four groups (n = 5) and treated as follows: G1: control; G2: transarterial embolization with Lipiodol; G3: transarterial embolization with CaPL; and G4: transarterial embolization with L-Arg@CaPL. At day 0, the hepatic arteries of these rats were embolized with Lipiodol, CaPL, and L-Arg@CaPL after being subjected to MR imaging system. The embolized dosage of L-Arg, CaP, and Lipiodol were 1.4 mg, 2.0 mg, and 100 μL, respectively. At 3, 7, and 14 days post various treatments, these rats were intraperitoneally injected with commercial gadolinium contrast agent and then subjected to a 3.0-T MR imaging system for recording the tumor volumes. To further evaluate the therapeutic efficacies, one rat in each group was sacrificed for their tumors collected and cryosectioned for H&E and TUNEL staining on day 8.

To evaluate the effects of L-Arg@CaPL treatment on immunosuppressive TME, these tumors in rats after different treatments were resected for immunofluorescent staining of CD8^+^ T cells, NK cells, and Treg cells. To elucidate the mechanism of T cell metabolism regulated by L-Arg@CaPL, these spleens in rats after different treatments were resected to prepare single-cell suspensions. After being stained with corresponding fluorescent antibodies according to the vendors’ protocols, these single-cell suspensions were subjected to flow cytometry for analyzing intratumoral abundances of T_CM_, (CD8^+^CD45RC^−^). Additionally, the secretion levels of diverse cytokines including IL-2, IFN-γ, TNF-α, IL-10, and TGF-β1 in the supernatant of these tumor lysates were determined by using corresponding commercial ELISA kits following the vendors’ protocols.

To evaluate the safety and biocompatibility of our proposed strategies, healthy rats after TACE with L-Arg@CaPL were sacrificed at 30 days. The dosage of L-Arg, CaP, and Lipiodol were 1.4 mg, 2.0 mg, and 100 μL, respectively. The major organs were collected for H&E staining. Meanwhile, the blood samples were collected for blood biochemistry tests, including total bilirubin (TBIL), serum calcium (Ca^2+^), alanine aminotransferase (ALT), and serum creatinine (CREA).

**Statistic**

The tests were performed at least three times independently. All quantitative data are presented as the mean ± SD. Statistical analyses were conducted using GraphPad Prism. The p values were calculated by one-way analysis of variance (ANOVA), ***p < 0.001, **p < 0.01, and *p < 0.05.

**Figure S1.** The hydrodynamic sizes of CaP NPs (n = 3).

**Figure S2.** XRD spectrum of CaP NPs.

**
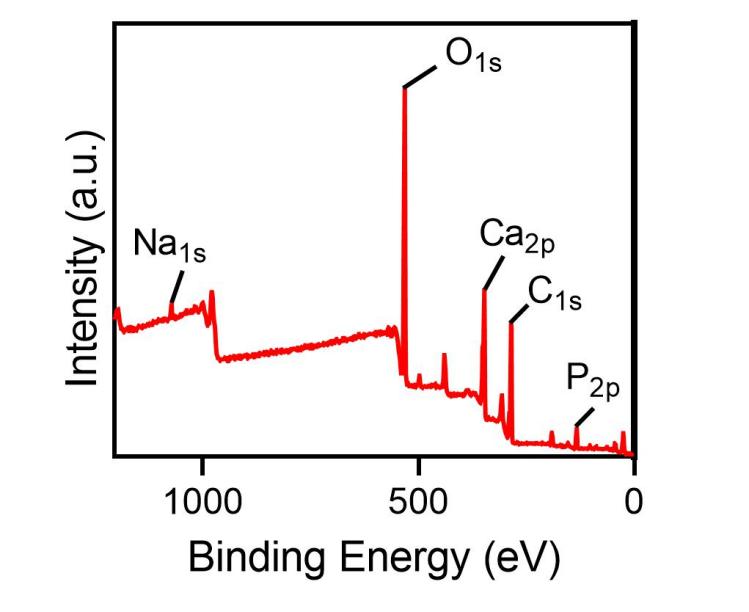
**

**Figure S3.** XPS spectra of CaP NPs.

**Figure S4.** Hemolysis assay of CaP NPs (n = 3).

**
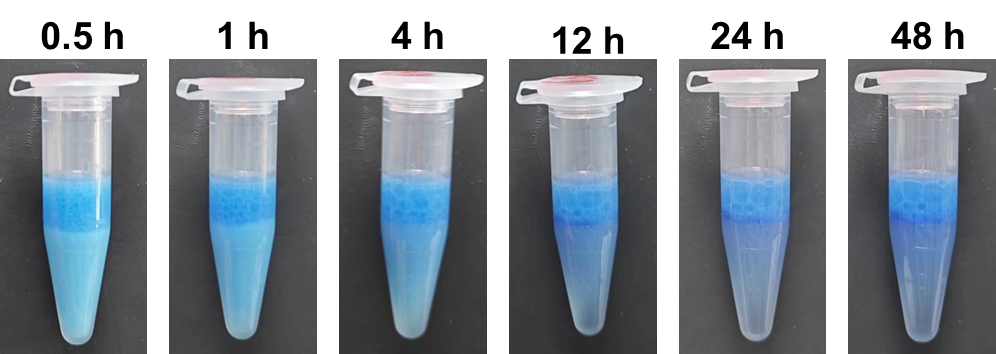
**

**Figure S5.** Time-dependent digital photographs of Lipiodol-water emulsions (methylene blue dissolved in aqueous-phase).

**
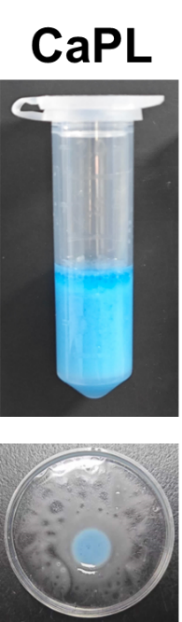
**

**Figure S6.** Representative optical images of CaPL at 24 h post preparation (top) and corresponding emulsions added to water (bottom).

**
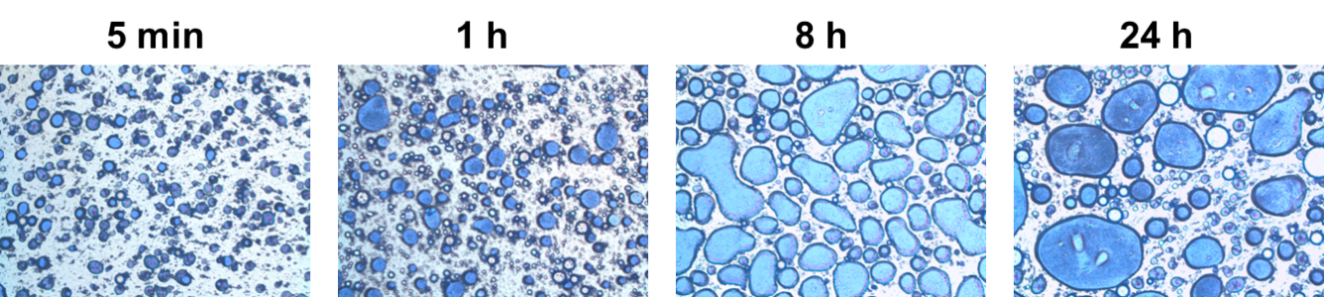
**

**Figure S7.** Time-dependent optical microscopic images of CaPL.

**
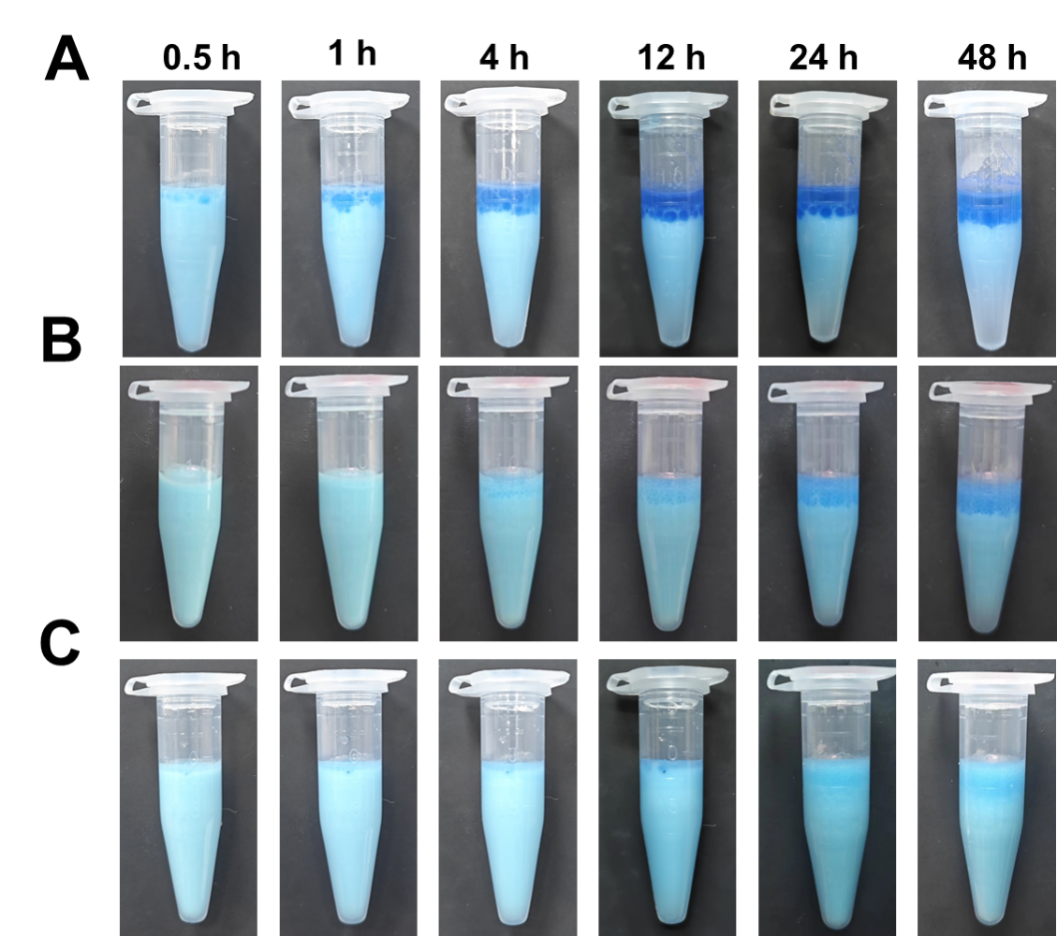
**

**Figure S8.** Time-dependent digital photographs of CaPL stabilized by CaP NPs at a dose of 3.3 mg (A), 6.6 mg (B), and 13.2 mg (C), respectively.

**
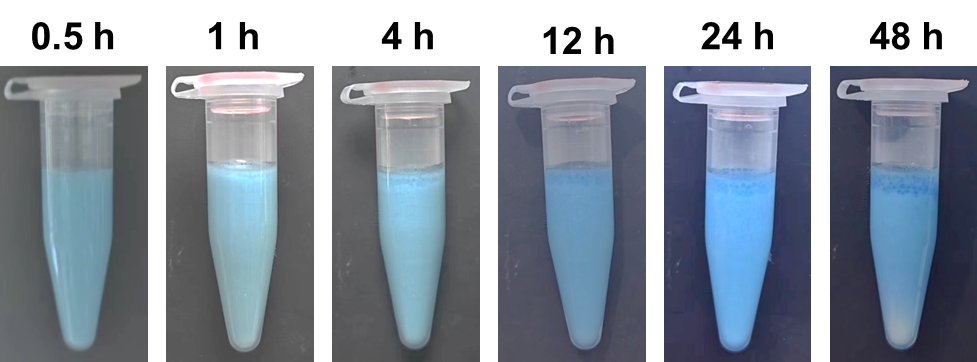
**

**Figure S9.** Time-dependent digital photographs of L-Arg@CaPL stabilized by CaP NPs at a dose of 10 mg.

**Figure S10.** Spare respiratory capacity (SRC) of T cells following different treatments as indicated (n = 3). Data are presented as mean values ± SD. One-way analysis of variance (ANOVA) was used for multiple comparisons.

**
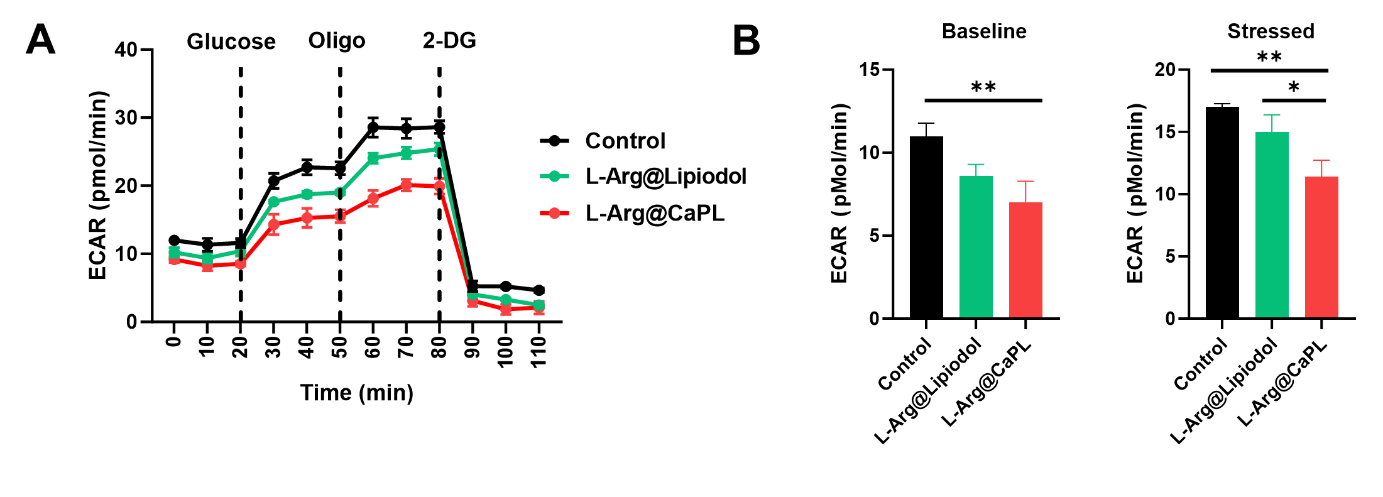
**

**Figure S11.** (A) Analyzes of extracellular acidification rate (ECAR) of T cells after different treatments. (B) ECRA quantifications of basic glycolytic ability and maximal glycolytic ability (n = 3). Data are presented as mean values ± SD. One-way analysis of variance (ANOVA) was used for multiple comparisons.


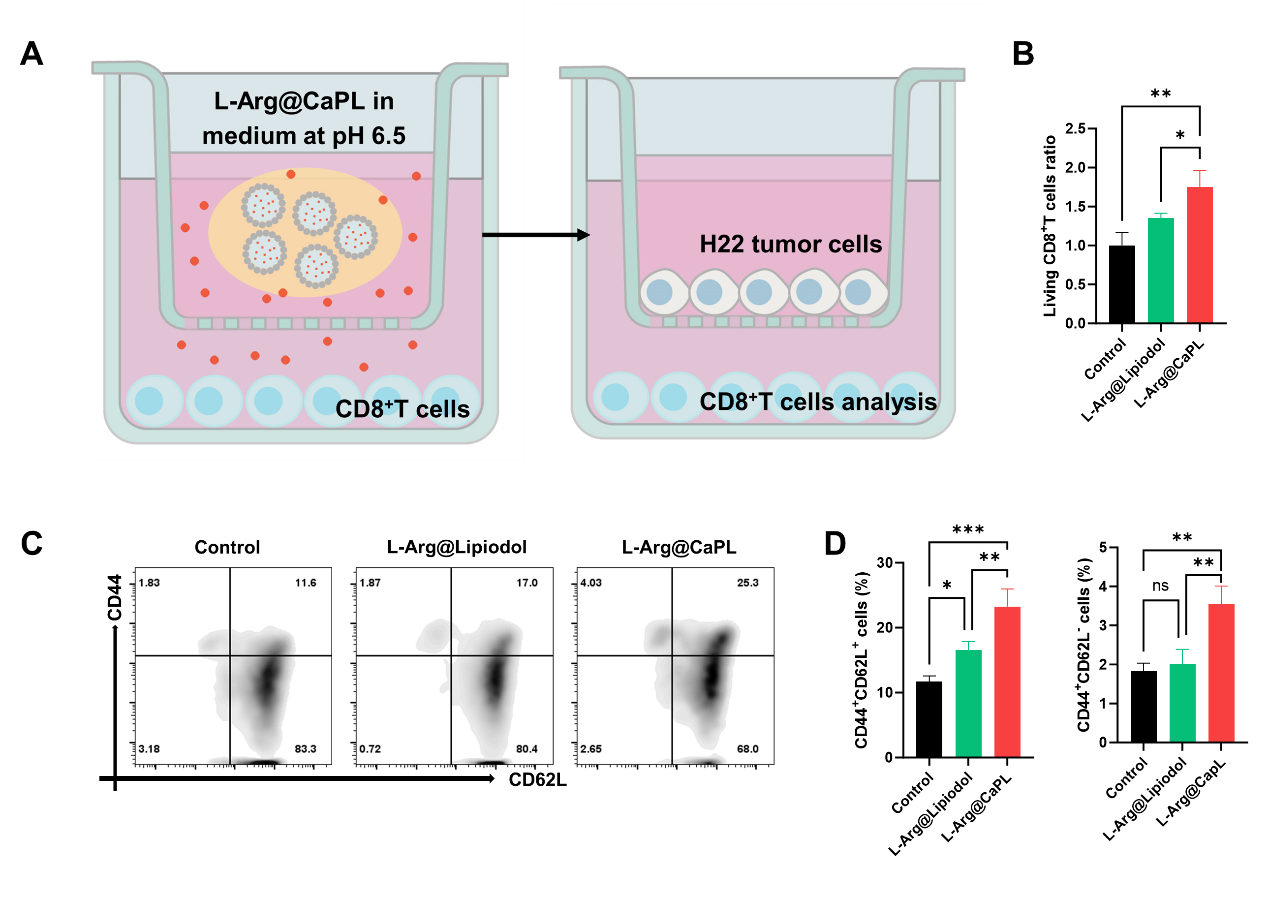


**Figure S12.** (A) A schematic diagram illustrating in vitro co-culture experiments for the evaluation of T cell functionality. (B) The relative proliferative capacity of T cells. (C-D) Representative flow cytometric analysis and quantification results of the frequencies of central memory T cells (T_CM,_ CD44^+^CD62L^−^) and effector memory T cells (T_EM,_ CD44^+^CD62L^−^) (n=3). Data are presented as mean values ± SD. One-way analysis of variance (ANOVA) was used for multiple comparisons.

**
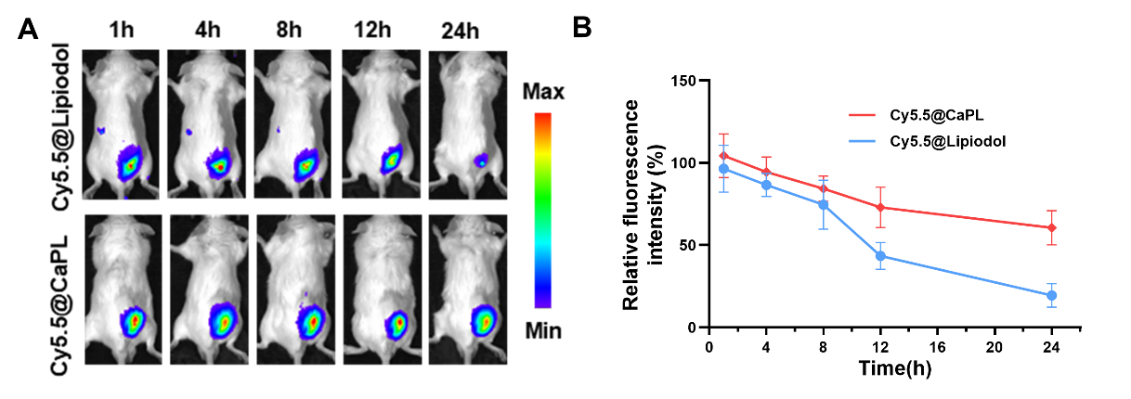
**

**Figure S13.** (A) *In vivo* fluorescence imaging of H22 tumor-bearing mice post intratumoral injection of Cy5.5@lipiodol, and Cy5.5@CaPL at the indicated time points. (B) Relative fluorescence intensity of H22 tumor area based on the images shown in A.

**
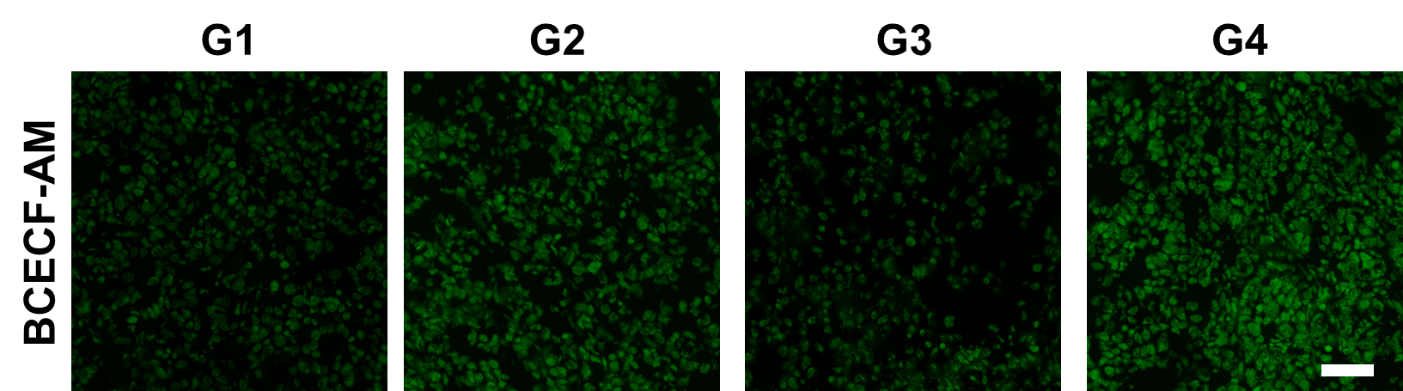
**

**Figure S14.** Fluorescence images of tumor slices after BCECF-AM staining. Scale bar: 50 μm.

**Figure S15.** The alterations in ATP levels within CD8^+^ T cells following various interventions as indicated (n = 3). Data are presented as mean values ± SD. One-way analysis of variance (ANOVA) was used for multiple comparisons.


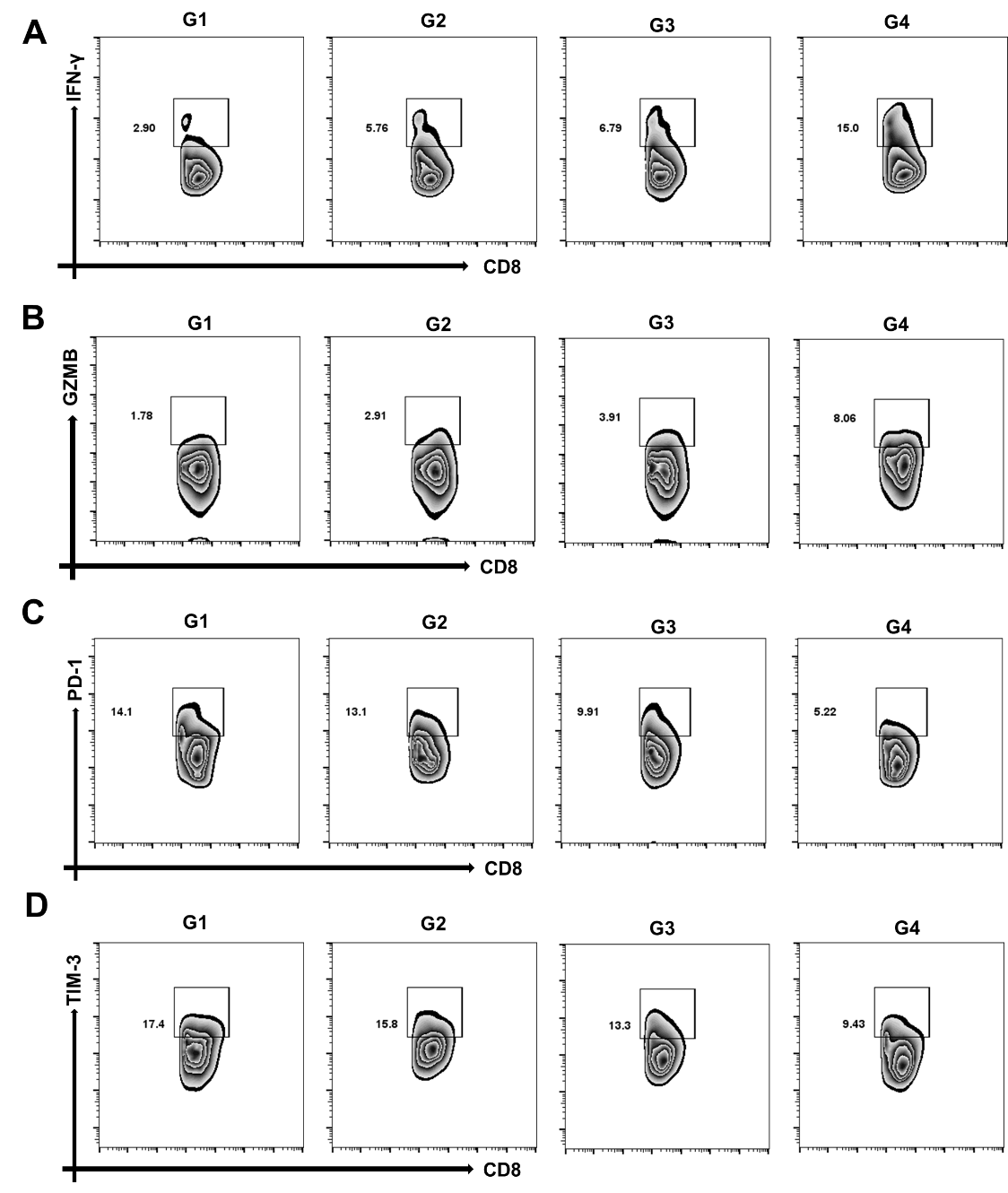


**Figure S16.** Representative flow cytometric analysis of the frequencies of effector CD8^+^ T cells (IFN-γ^+^CD8^+^, GZMB^+^CD8^+^) and exhausted CD8^+^ T cells (PD-1^+^CD8^+^, TIM-3^+^CD8^+^)


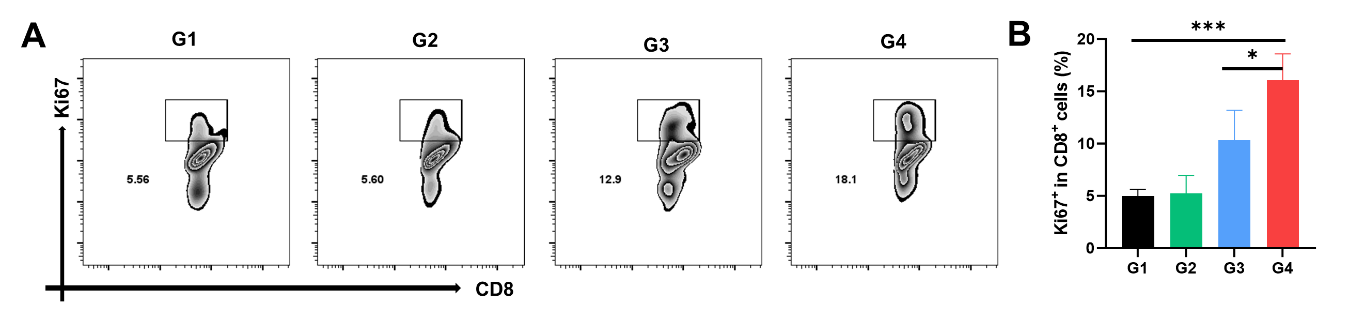


**Figure S17.** Representative flow cytometric analysis and quantification results of the frequencies of Ki67^+^ in CD8^+^ T cells (n = 3). Data are presented as mean values ± SD. One-way analysis of variance (ANOVA) was used for multiple comparisons.


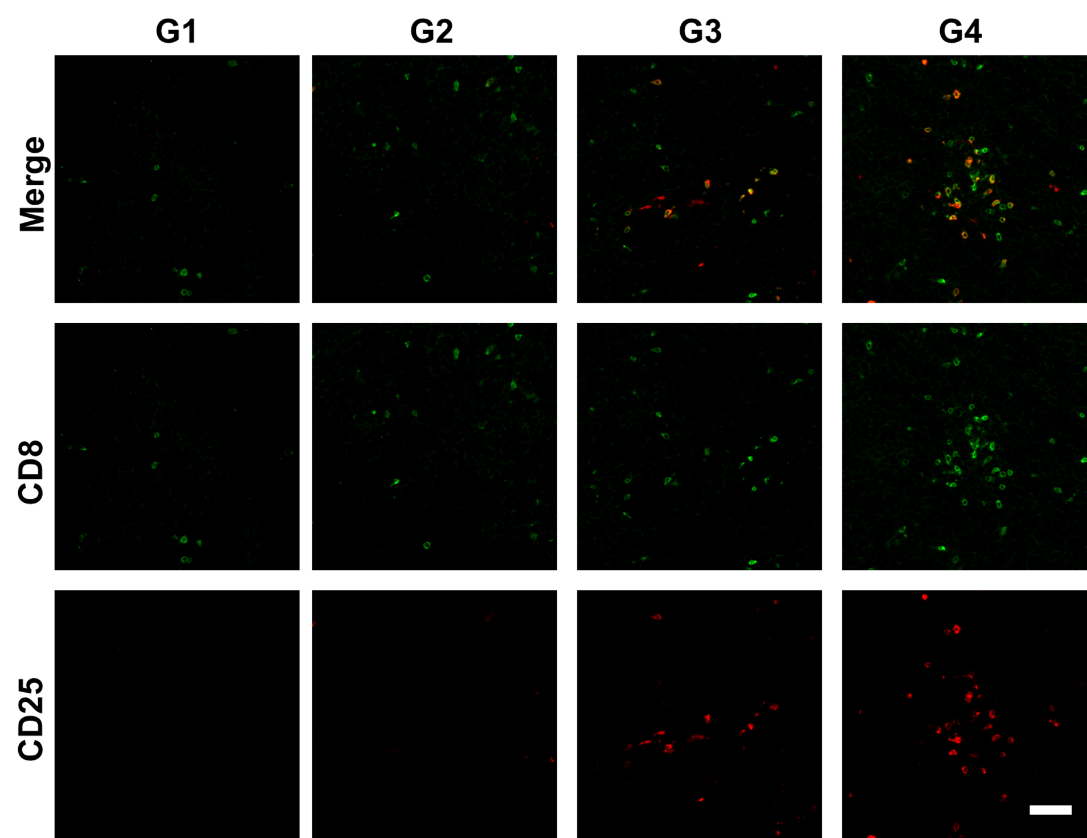


**Figure S18.** Immunofluorescent-stained images showing the infiltration of activated CD8^+^ T cells, within H22 tumors. Scale bar: 50 µm.

**
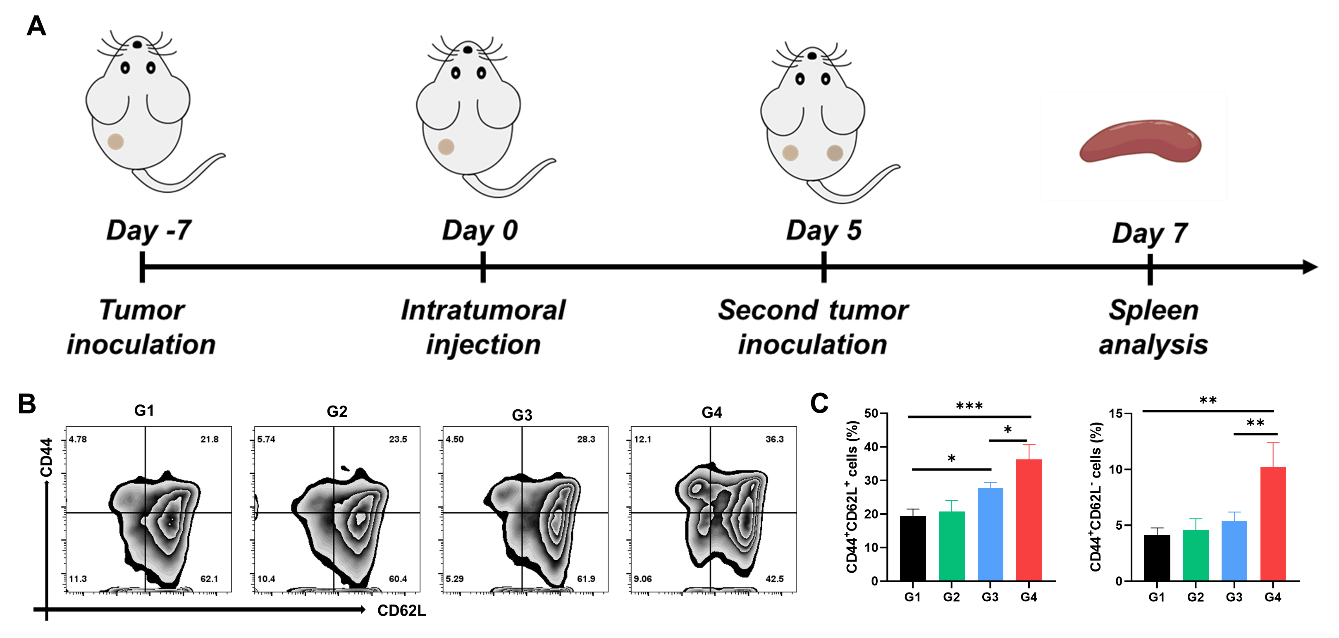
**

**Figure S19.** (A) A schematic diagram of the therapeutic schedule. (B-C) Representative flow cytometric analysis and quantification results of the frequencies of T_CM_ (CD44^+^CD62L^+^) and T_EM_ (CD44^+^CD62L^−^) (n=3). Data are presented as mean values ± SD. One-way analysis of variance (ANOVA) was used for multiple comparisons.

**Figure S20.** Secretion levels of TNF-α in the tumors of mice post varying treatments as indicated (n = 3). Data are presented as mean values ± SD. One-way analysis of variance (ANOVA) was used for multiple comparisons.

**
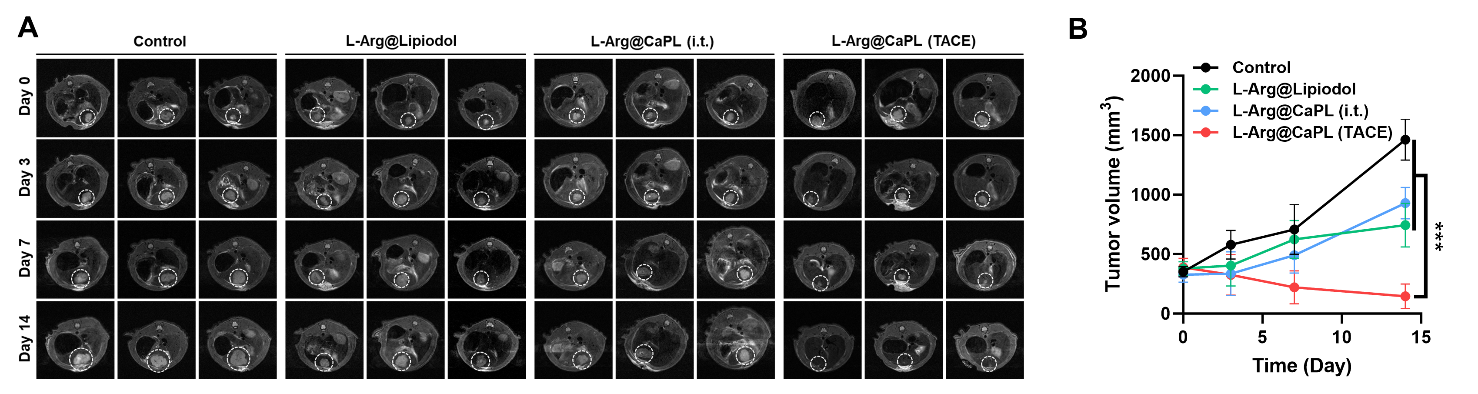
**

**Figure S21.** (A) Representative T2 contrast-enhanced MR scanning of N1S1-bearing rats with different treatments as indicated. (B) Average tumor growth curves of different groups of N1S1 tumor-bearing rats after various treatments as indicated (n = 5). Data are presented as mean values ± SD. One-way analysis of variance (ANOVA) was used for multiple comparisons.

**
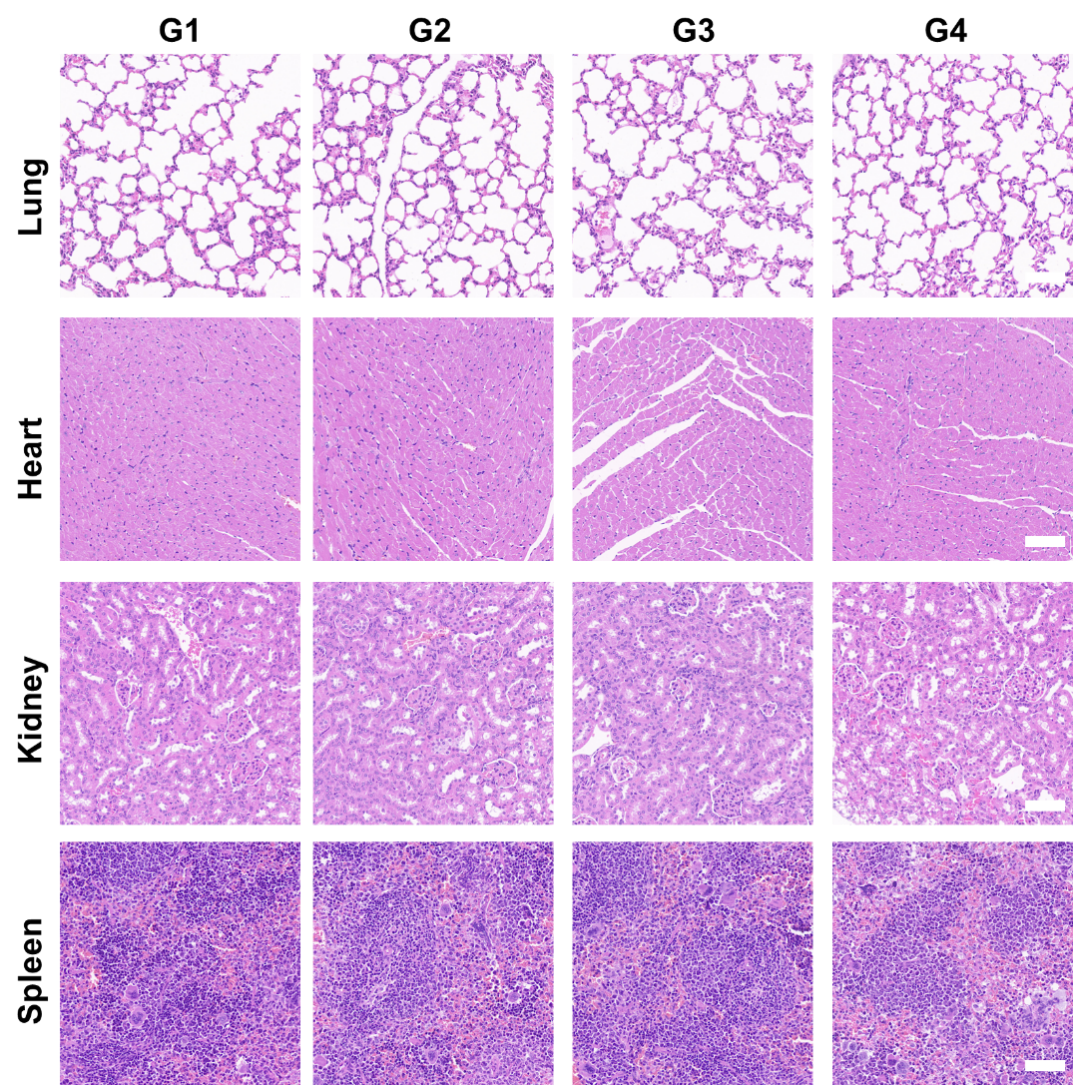
**

**Figure S22.** Hematoxylin and eosin (H&E) staining of rat major organs (lung, heart, kidney, and spleen) to examine the histological changes after different treatments. Scale bar: 200 µm.

**
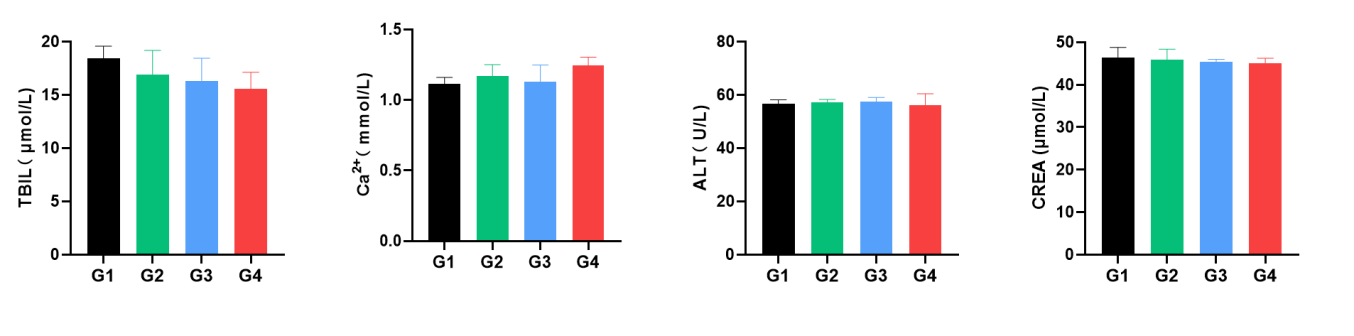
**

**Figure S23.** Blood biochemistry data of rats after different treatments. The measured indexes included total bilirubin (TBIL), serum calcium (Ca^2+^), and serum creatinine (CREA) (n=3).

**Figure S24.** The alterations in ATP levels within CD8^+^ T cells following various interventions as indicated (n = 3). Data are presented as mean values ± SD. One-way analysis of variance (ANOVA) was used for multiple comparisons.

**
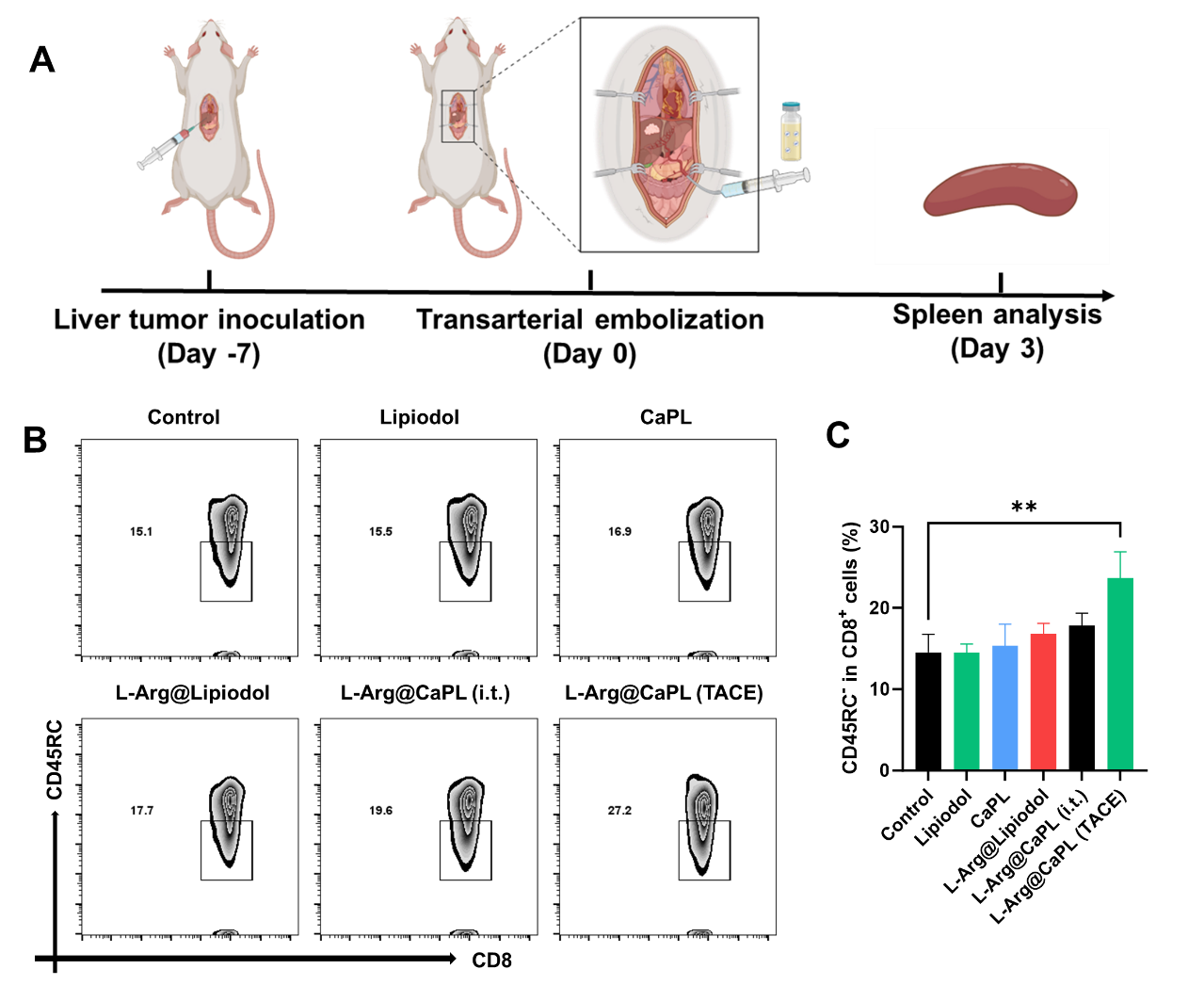
**

**Figure S25.** (A) A schematic diagram of the therapeutic schedule. (B-C) Representative flow cytometric analysis and quantification results of the frequencies of T_CM_ (n = 3). Data are presented as mean values ± SD. One-way analysis of variance (ANOVA) was used for multiple comparisons.

**Figure S26.** Secretion levels of TNF-α in the tumors of rats post varying treatments as indicated (n = 3). Data are presented as mean values ± SD. One-way analysis of variance (ANOVA) was used for multiple comparisons.
